# Brain spontaneous fluctuations in sensorimotor regions were directly related to eyes open and eyes closed: evidences from a machine learning approach

**DOI:** 10.3389/fnhum.2014.00645

**Published:** 2014-08-20

**Authors:** Bishan Liang, Delong Zhang, Xue Wen, Pengfei Xu, Xiaoling Peng, Xishan Huang, Ming Liu, Ruiwang Huang

**Affiliations:** ^1^Guangdong Key Laboratory of Mental Health and Cognitive Science, Center for Studies of Psychological Application, School of Psychology, South China Normal UniversityGuangzhou, China; ^2^Department of Radiology, Guangdong Province Hospital of Traditional Chinese MedicineGuangzhou, China; ^3^Guangzhou University of Chinese Medicine Postdoctoral Mobile Research StationGuangzhou, China; ^4^State Key Laboratory of Cognitive Neuroscience and Learning, Beijing Normal UniversityBeijing, China

**Keywords:** resting-state fMRI, fractional amplitude of low-frequency fluctuation (fALFF), support vector machine (SVM), eyes closed, eyes open

## Abstract

Previous studies have demonstrated that the difference between resting-state brain activations depends on whether the subject was eyes open (EO) or eyes closed (EC). However, whether the spontaneous fluctuations are directly related to these two different resting states are still largely unclear. In the present study, we acquired resting-state functional magnetic resonance imaging data from 24 healthy subjects (11 males, 20.17 ± 2.74 years) under the EO and EC states. The amplitude of the spontaneous brain activity in low-frequency band was subsequently investigated by using the metric of fractional amplitude of low frequency fluctuation (fALFF) for each subject under each state. A support vector machine (SVM) analysis was then applied to evaluate whether the category of resting states could be determined from the brain spontaneous fluctuations. We demonstrated that these two resting states could be decoded from the identified pattern of brain spontaneous fluctuations, predominantly based on fALFF in the sensorimotor module. Specifically, we observed prominent relationships between increased fALFF for EC and decreased fALFF for EO in sensorimotor regions. Overall, the present results indicate that a SVM performs well in the discrimination between the brain spontaneous fluctuations of distinct resting states and provide new insight into the neural substrate of the resting states during EC and EO.

## Introduction

The modulation of resting-state brain activity has attracted great attention in recent years (Hüfner et al., 2009), and previous studies have demonstrated that resting-state brain activity is differentially affected based on whether the subjects have their eyes open (EO) or eyes closed (EC). Until recently, the widespread influences of EO versus EC on resting-state activity have been demonstrated using methods ranging from regional spontaneous activity (Marx et al., 2003, 2004; Mcavoy et al., 2008; Bianciardi et al., 2009) to functional connectivity (Yan et al., 2009; Zou et al., 2009; Wu et al., 2010; Mcavoy et al., 2012) and to network topological organization (Jao et al., 2013; Xu et al., 2014). Despite these advances, little is known about whether there are spontaneous fluctuations that are directly related to eye behavior states.

Several functional magnetic resonance imaging (fMRI) studies have already shown that spontaneous brain oscillations are different between resting states with EC and EO in many regions, including the visual cortex (Raichle et al., 2001; Uludag et al., 2004), auditory cortex (Marx et al., 2003; Qin et al., 2013), somatosensory cortex, fronto-parietal attentional regions, and default mode network (DMN) (Yan et al., 2009). Importantly, alterations of spontaneous activity associated with different eye behavior states vary between EC and EO. For example, our recent study (Xu et al., 2014) found that the attentional, ocular motor, and arousal systems showed higher regional nodal properties (e.g., efficiency, degree, and betweenness centrality) in EO than in EC; in contrast, the visual, auditory, and somatosensory systems and parts of the DMN (Raichle et al., 2001) showed significantly lower regional nodal properties in EO than in EC. These findings may confirm the existence of an “exteroceptive” mental state, characterized by attentional and ocular motor activity during EO, and an “interoceptive” mental state, characterized by imagination and multisensory activity during EC (Marx et al., 2004). It should be noted that the notion of exteroceptive and interoceptive mental states is derived from the direct measurement of spontaneous brain oscillations (Marx et al., 2004). In addition, alterations in spontaneous activity (e.g., amplitude of low frequency fluctuation, ALFF; fractional ALFF, fALFF) between the EO and EC states have been found in the visual cortex, paracentral lobule (PCL) (Yang et al., 2007), middle temporal gyrus, anterior insula, and DMN regions (Yan et al., 2009). Although the widely distributed differences of brain activity are related to these two mental states, the knowledge whether the spontaneous fluctuations in these brain regions could combine to be directly related to the two states is still largely limited. Thereby, a sophisticated approach is consequently necessary to assess the link of the spontaneous fluctuations to the eye behavior states, which will promote the investigation of fundamental neural differences between EC and EO.

Recently, machine learning approach has been widely applied to investigate the relationship between the distributed functional signal and the brain states (Craddock et al., 2009; Poldrack et al., 2009; Dosenbach et al., 2010; Shen et al., 2010; Naselaris et al., 2011; Zeng et al., 2012; Vergun et al., 2013). By building a mathematical model (e.g., support vector machine, SVM), we could predict the brain states from the spatially distributed spontaneous activities and further assess their relationship directly. In the present study, we sought to identify whether the spontaneous fluctuation amplitude directly link to the EC and EO by using the SVM approach. Toward this end, we recruited 24 healthy subjects and collected their resting state fMRI data. By using fALFF, we first localized those brain regions showing differences in spontaneous resting-state activity between EC and EO. Next, a machine learning method was further applied to examine whether there exists a pattern of the spontaneous fluctuation amplitude which is directly related to the eye behavior states.

## Materials and methods

### Subjects

A total of 24 right-handed healthy undergraduates/postgraduates (11 males/13 females, 20.17 ± 2.74 years) were recruited for the present study. No subjects had a history of neurological or psychiatric disorders or head injury. This study was approved by the Institutional Review Board of Beijing Normal University Imaging Center for Brain Research. Written informed consent was obtained from each subject before the experiment.

### Data acquisition

All MRI data were acquired on a 3T Siemens Trio TIM MR scanner powered with a total imaging matrix (TIM) technique at the Imaging Center for Brain Research, Beijing Normal University. The resting-state functional magnetic resonance imaging (rsfMRI) data were acquired using a 12-channel phased array receiver-only head coil. The rsfMRI data were obtained using a gradient-echo EPI sequence with the following two parameter sets: (A) *TR* = 2000 ms, *TE* = 30 ms, 33 transverse slices, slice thickness = 3.5 mm, gap = 0.7 mm, flip angle = 90°, FOV = 224 mm × 224 mm, matrix = 64 × 64, and 240 volumes covering the whole brain; (B) *TR* = 3000 ms, 40 transverse slices, slice thickness = 3.5 mm, no gap, 160 volumes, and the other parameters identical to those of (A). For each parameter set, all subjects underwent the rsfMRI scan under both conditions (EC and EO) in turn. Thus, there were four types of rsfMRI scan: AO, AC, BO, and BC. For each subject, all four scans were performed in the same session. The order of data acquisition was counterbalanced across all subjects. In addition, we also acquired high-resolution 3D brain structural images for each subject by using MP-RAGE sequence with the implementation of parallel imaging scheme GRAPPA (GeneRalized Autocalibrating Partially Parallel Acquisitions) (Griswold et al., 2002) and the acceleration factor 2.

### Data preprocessing

For the present study, we analyzed the rsfMRI data corresponding to *TR* = 2000 ms only. Data preprocessing was performed using DPARSF (http://www.restfmri.net/forum/DPARSF) with SPM8 (http://www.fil.ion.ucl.ac.uk/spm/). We discarded the first 10 volumes of the functional images to account for signal equilibrium and the subjects' adaptation to the scanning environment. We then corrected the remaining functional images for within-scan acquisition time differences between slices and realigned all images to the first volume to correct for head motion. This realignment procedure provided us with a record of head motion during each rsfMRI scan. None of the subjects were excluded for excessive head motion based on criteria of >2 mm displacement or an angular rotation of >2° in any direction. The summary scalars of both gross (maximum and root mean square) and micro (mean frame-wise displacement) head motion were matched between the two conditions (all *p* > 0.14). The corrected functional images were subsequently spatially normalized to the MNI standard template using an optimum 12-parameter affine transformation and non-linear deformations and were re-sampled to a voxel size of 3 × 3 × 3 mm^3^. Finally, the linear trend of the fMRI data was estimated with a least-square fitting of a straight line and was removed from the functional data.

### Calculation of fALFF

We calculated the fALFF in a voxel-wise manner following the procedures described in previous studies (Zang et al., 2007; Zou et al., 2008; Song et al., 2011). After data preprocessing, we transformed the data time series to the frequency domain using fast Fourier transformation (FFT), obtained the power spectrum, and then averaged across 0.01–0.08 Hz at each voxel. The power of a given frequency is proportional to the square of the amplitude of this frequency component in the original time series in the time domain. This averaged square root was defined as the ALFF. Next, the fALFF was computed as the ratio of the ALFF in the range of 0.01–0.08 Hz to that of the entire frequency range (0–0.25 Hz) for each voxel. The fALFF map was smoothed (full width at half maximum = 6 mm) and the normalized fALFF map was obtained by dividing fALFF at each voxel by each individual's global mean fALFF value.

### Individual fALFF maps

Using an existing template that was functionally defined based on a meta-analysis in a previous study (Dosenbach et al., 2010), we defined 160 cortical regions of interest (ROIs) for the entire cortex (Table S1). This template broadly covers most of the cortex and cerebellum and has been used in several studies, such as brain functional network studies (Wang et al., 2011; Hwang et al., 2013). We first extracted the fALFF value at each voxel for each resting condition (EO or EC). We then calculated the mean fALFF for each ROI by averaging the fALFF values across all voxels for the selected ROI in each subject. Finally, we divided the 160 ROIs into six modules (default network, fronto-parietal, cingulo-opercular, sensorimotor, occipital, and cerebellum) according to a meta-analysis of cognitive experimental fMRI studies (Dosenbach et al., 2010). Thus, individual fALFF maps were constructed, and fALFF values for the 160 ROIs were obtained for each resting condition.

### Machine learning method

**Figure 1:**
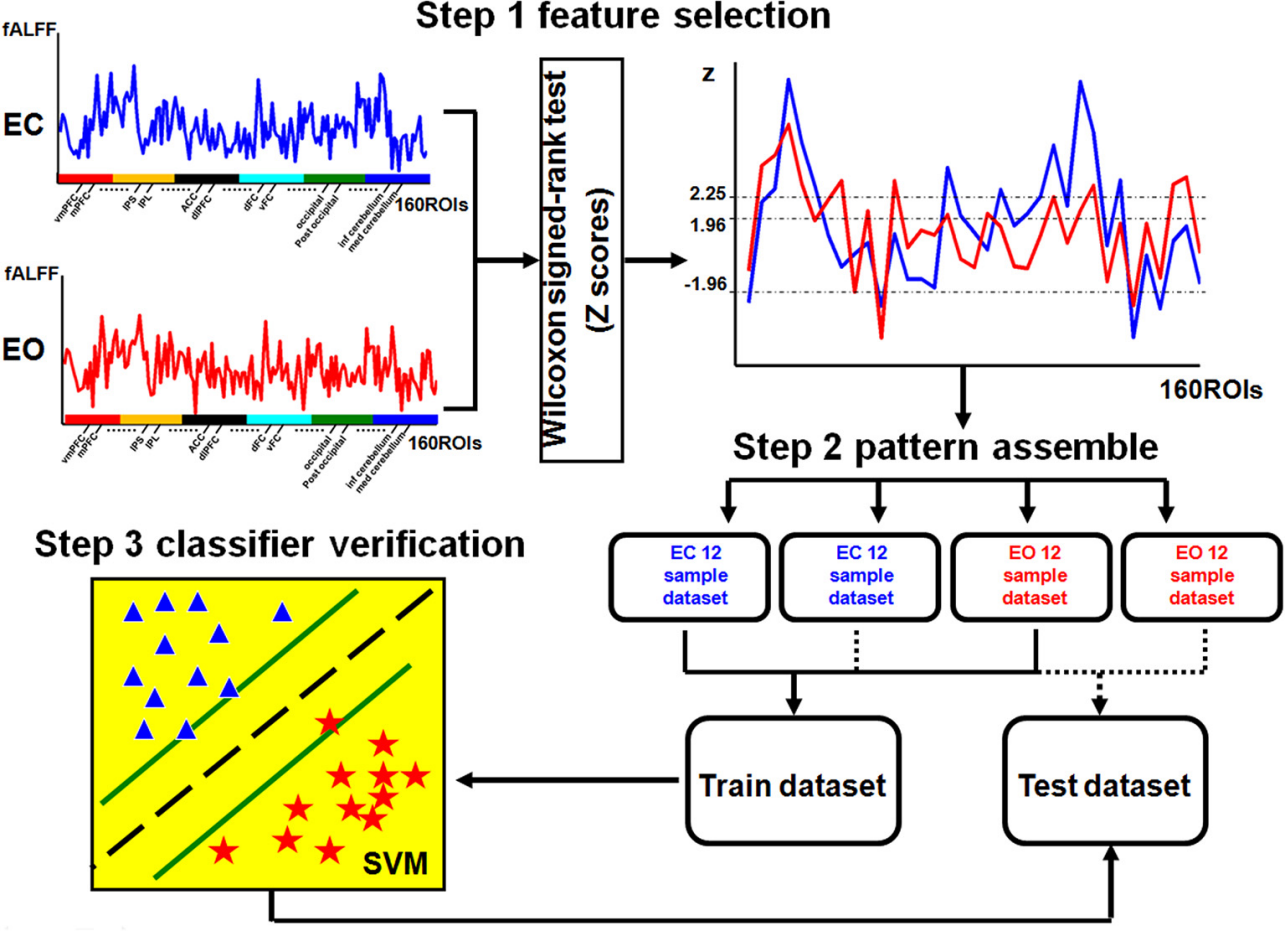
**Flowchart of MVPA**. The fALFF values of 160 ROIs were used as the features. A nonparametric two-sample Wilcoxon signed-rank test was performed to detect discriminatory patterns between two conditions (EC and EO). The selected features from each of the 12 subjects for each condition were ranked as a sample dataset, which resulted in two sample datasets for each condition: one was used to construct the training dataset (24 data samples across two conditions) and another one for the testing dataset (24 data samples across two conditions). The SVM classifier was trained with the training dataset, and the performance of the trained classifier was tested with the testing dataset. In step 3, stars and triangles represent the samples of the two conditions.

The LIBSVM toolbox (http://www.csie.ntu.edu.tw/~cjlin/libsvm/) with the linear support vector classification (SVC) was applied as the classifier for the multivariate pattern analysis (MVPA) (Zhang et al., 2013). The flowchart of the analysis stream for the MVPA method is shown in Figure 1.

The first step was feature selection. To reduce the dimensionality of the discriminatory patterns, we selected features using a nonparametric two-sample Wilcoxon signed-rank test (Chen et al., 2011). First, we calculated the mean fALFF for each ROI by averaging across all subjects in two resting conditions (EO and EC) separately. Then, the differences among all the features, represented by the mean fALFF value for each ROI (a total of 160 features) between the two resting conditions (EO and EC), were estimated to have a normal distribution and were selected as the discriminatory features for MVPA.

To extract highly discriminatory brain areas, we used four rankings of the difference scores (Z-scores) of the Wilcoxon signed-rank test as the criteria for the feature selection: |*Z*| > 1.96 (−0.05<p < 0.05), *Z* > 1.96 (*p* < 0.05), *Z* > 2.25 (*p* < 0.01), and *Z* < −1.96 (*p* > −0.05). Using these four criteria, we were able to systematically decrease the number of different regions in the fALFF maps between the two conditions. The selected features were the same for all subjects based on different Z criteria.

The second step was pattern assembly. Four pattern types were identified from the selected discriminatory features according to different criteria. For each discriminatory pattern, we ranked the features of each subject for each resting condition (EO or EC) in a row vector to constitute the sample data, and individual rows were combined into a sample dataset. For each of the 24 subjects, the rsfMRI scan was performed under two resting conditions, EC and EO, resulting in a dataset containing 48 samples. Finally, the samples for each resting condition were randomly divided into two sets of 12 samples each. Half of the data was used to construct the training dataset (24 samples across two conditions), and the other half was used as the testing dataset (24 samples across two conditions). The aim was to improve the reliability of the classifier validation as much as possible.

The third step was classifier verification. The sample data in the training and testing datasets were labeled with category labels (EC = 1 and EO = −1). The SVC classifier was trained on the training dataset and the corresponding category labels, and the performance of the trained classifier was tested using the testing dataset and the corresponding category labels. Using a receiver operating characteristic (ROC) curve, we evaluated the performance of the trained classifier based on its accuracy which was estimated as the percentage of the correct predicted category label.

## Results

### fALFF in EO and EC

**Figure 2:**
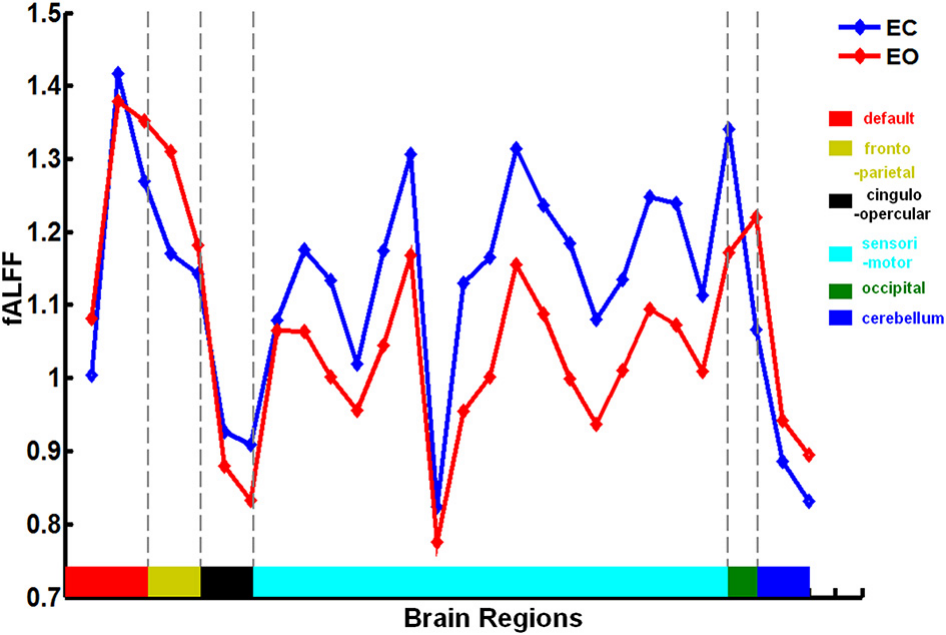
**Brain regions involved in the discriminative pattern corresponding to |*Z*| > 1.96**. The details of the brain regions and modules are listed in Table S1.

There was a significant difference in the mean fALFF between the two resting conditions, EC and EO, using a two-sample Wilcoxon signed-rank test (|*Z*| > 1.96; Figure 2). Compared with EO, the fALFF values in EC were significantly increased in the sensorimotor module and cingulo-opercular region but significantly decreased in the fronto-parietal cortex, occipital cortex, and cerebellum. Additionally, we noted that fALFF changes between EO and EC were far from consistent in the DMN regions. Compared with EO, the fALFF value in EC was higher in the left inferior parietal supramarginal gyrus (IPS. L), but lower in the left occipital cortex and ventromedial prefrontal cortex (vmPFC.L) (Table 1).

**Table 1:** **Brain regions involved in the discriminatory pattern with |*Z*| > 1.96**.

**Index**	**Selected regions**		**Coordinates**			**Modules**	***Z*-value**
			***x* (mm)**	***y* (mm)**	***z* (mm)**		
1	parietal	R	18	−27	62	sensorimotor	3.34
2	frontal	R	53	−3	32	sensorimotor	2.92
3	precentral gyrus	L	−54	−9	23	sensorimotor	2.92
4	parietal	L	−47	−12	36	sensorimotor	2.92
5	parietal	L	−55	−22	38	sensorimotor	2.92
6	post insula	R	42	−24	17	sensorimotor	2.92
7	parietal	L	−24	−30	64	sensorimotor	2.92
8	IPS	L	−36	−69	40	default	2.50
9	basal ganglia	R	11	−24	2	cingulo-opercular	2.50
10	mid insula	L	−36	−12	15	sensorimotor	2.50
11	parietal	L	−47	−18	50	sensorimotor	2.50
12	post parietal	L	−41	−31	48	sensorimotor	2.50
13	sup parietal	R	34	−39	65	sensorimotor	2.50
14	mid insula	R	37	−2	−3	cingulo-opercular	2.09
15	vFC	L	−55	7	23	sensorimotor	2.09
16	precentral gyrus	R	58	−3	17	sensorimotor	2.09
17	mid insula	L	−42	−3	11	sensorimotor	2.09
18	mid insula	R	33	−12	16	sensorimotor	2.09
19	temporal	R	59	−13	8	sensorimotor	2.09
20	parietal	R	41	−23	55	sensorimotor	2.09
21	temporal	L	−53	−37	13	sensorimotor	2.09
22	occipital	L	−42	−76	26	default	−2.09
23	aPFC	L	−29	57	10	fronto-parietal	−2.09
24	dlPFC	L	−44	27	33	fronto-parietal	−2.09
25	post occipital	R	29	−81	14	occipital	−2.09
26	inf cerebellum	L	−21	−79	−33	cerebellum	−2.09
27	vmPFC	R	−36	−12	15	sensorimotor	−2.50
28	inf cerebellum	L	−47	−18	50	sensorimotor	−2.50

The (x, y, z) coordinates correspond to the peak voxel in MNI space. The Z-value indicates the difference scores, which were calculated from the nonparametric two-sample Wilcoxon signed-rank test, and a positive value indicates that the Z-value of EC was higher than that of EO (and vice versa). Six modules were evaluated, including default, fronto-parietal, cingulo-opercular, sensorimotor, occipital, and cerebellum. L (R), left (right) hemisphere.

### SVM classifier performance

**Figure 3:**
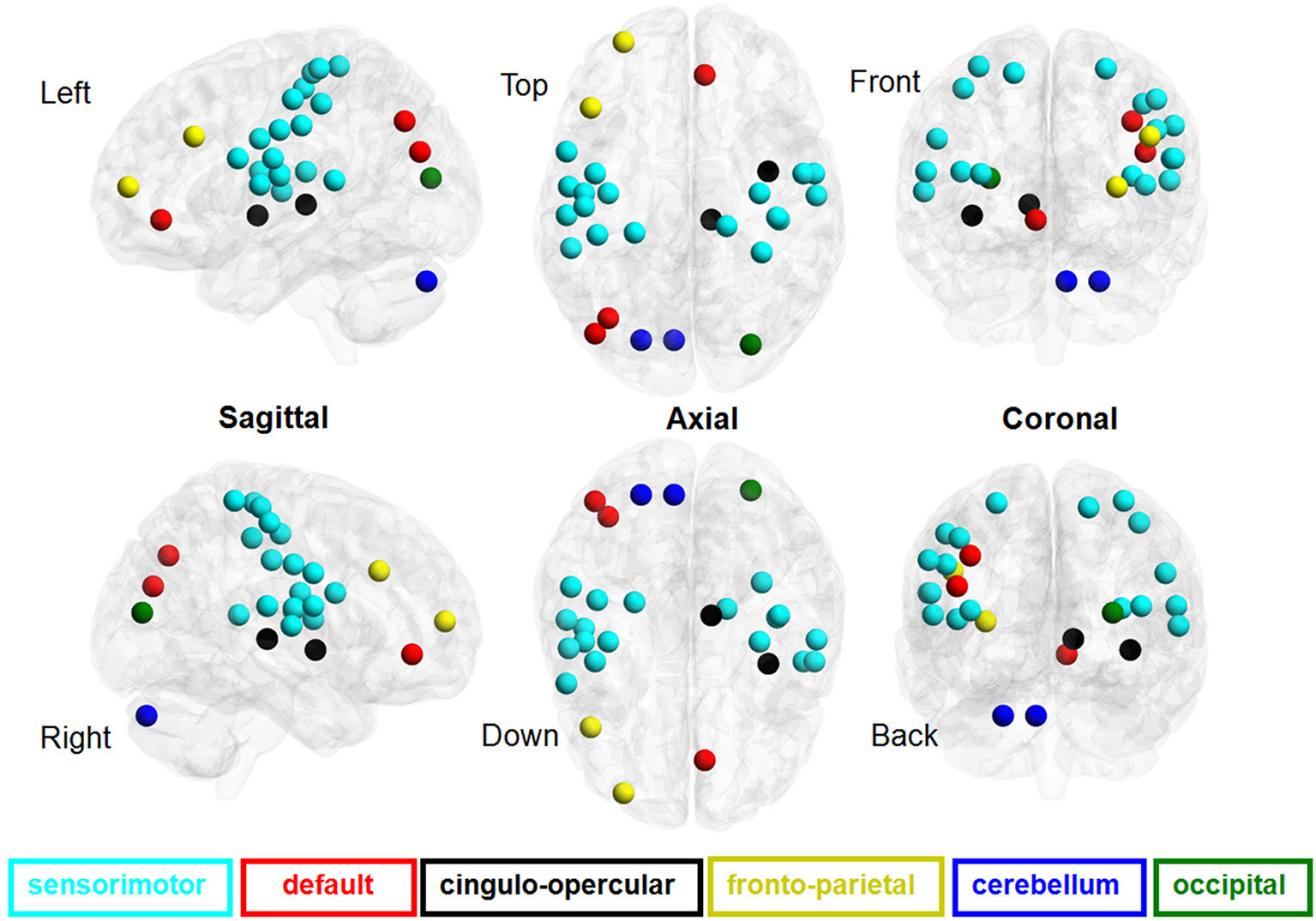
**ROC curves of the performance of SVM classifiers corresponding to different neural fALFF patterns**. The yellow line corresponds to the patterns of brain regions with *Z* < −1.96, the blue line to *Z* > 1.96, the green line to *Z* > 2.25, and the red line to |*Z*| > 1.96.

We found that the eye conditions could be decoded from the brain fALFF map using a SVM composed of 28 discriminatory brain regions (|*Z*| > 1.96) (Figure 3). The accuracy of this classifier was 85% (Table 1).

Using a subset of brain regions, the accuracy of the classifier was 84% (21 brain regions with *Z* > 1.96) and 88% (13 brain regions with *Z* > 2.25). However, the classifier showed poor performance (accuracy = 63%) based on a pattern of 7 brain regions (*Z* < −1.96), i.e., the fALFF of EC was significantly lower than that of EO.

**Figure 4:**
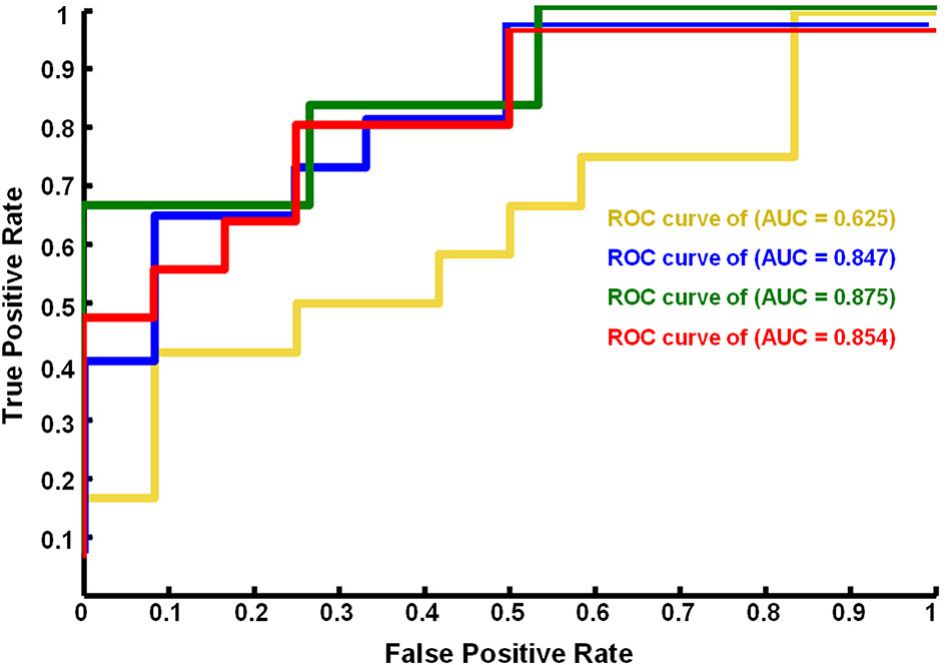
**Mean fALFF values of each brain region in the neural fALFF map with |*Z*| > 1.96 related to the EC and EO states**. fALFF was calculated as the ratio of the ALFF value in the range of 0.01–0.08 Hz to that of the entire frequency range (0–0.25 Hz). The brain regions and corresponding modules are same to those in Figure 2.

Figure 4 shows the ROC curve of the neural activity patterns associated with different brain regions (|*Z*| >1.96, *Z* > 1.96, *Z* < −1.96, and *Z* > 2.25). This figure clearly demonstrates that the pattern of brain regions with *Z* > 1.96 was similar to the pattern of regions with |*Z*| > 1.96 in decoding the eye conditions.

For *Z* > 1.96, the brain regions were widely distributed across the sensorimotor module, DMN, and cingulo-opercular region, with nearly 86% of these brain regions located in the sensorimotor module. For *Z*< −1.96, however, the brain regions were mainly located in the DMN, fronto-parietal cortex, occipital cortex, and cerebellum.

## Discussion

The present study investigated whether the fALFF could be directly related to distinct resting conditions (i.e., EC and EO) using a SVM. The main findings can be summarized as follows: (i) compared with EO, the EC condition induced higher fALFF values primarily in the sensorimotor cortex and cingulo-opercular region but lower fALFF values in the fronto-parietal cortex, occipital cortex, and cerebellum; (ii) the eye conditions could be decoded from the fALFF of the sensorimotor regions using a SVM; and (iii) increased or decreased fALFF in the sensorimotor cortex was directly related to the EC and EO conditions, respectively.

### fALFF of different resting states

The present study demonstrated that changes in fALFF were spatially distributed and predominantly located in the posterior occipital cortex, DMN-associated regions (e.g., intraparietal sulcus [IPS]), fronto-parietal regions, sensorimotor cortex, cerebellum, basal ganglia, and middle insula (Table 1). These results are consistent with previous studies (Yang et al., 2007; Yan et al., 2009; Jao et al., 2013), which indicated that the ALFF can reflect different cerebral physiological states (Yang et al., 2007; Zang et al., 2007). Furthermore, differences in the ALFF between the EC and EO states have previously been identified in regions such as the right PCL, visual cortex (Yang et al., 2007), and regions associated with the DMN (e.g., posterior cingulate cortex [PCC] and medial prefrontal cortex [MPFC]; (Yan et al., 2009).

Deactivation of the DMN is well known to account for a substantial portion of resting-state brain activity (Raichle et al., 2001; Fox et al., 2005; Fransson, 2005), and researchers have shown that neural activities in the DMN, one aspect of functional connectivity, were similar across different resting states such as EC and EO (Fox et al., 2005; Fransson, 2005). Accordingly, Yang et al. (2007) found no significant differences in the ALFF between the EC and EO states in the PCC, which is a key region of the DMN. These findings supported the conclusion that the DMN was not easily disrupted by visual information compared with the visual cortex (Greicius et al., 2003).

However, there have been discrepant findings regarding the neural activity of the DMN. For example, Yan et al. (2009) found that EO induced higher functional connectivity in the DMN and increased ALFF compared with EC. This observation suggests that increases in spontaneous neural activity may be related to greater involvement of non-specific neural activity during EO (e.g., non-goal-directed visual information gathering and evaluation or mind-wandering and daydreaming). Using nonparametric methods, our present data indicate that changes in the activity of DMN regions were inconsistent between the EC and EO states. We found that the IPS showed higher fALFF during EC whereas some parts of the occipital cortex showed lower fALFF during EC (Table 1). This finding may suggest the existence of differential responses of different DMN regions between the EC and EO states, and the identification of these DMN changes may depend on specific statistical methods.

Similarly, there are also contradictory views regarding spontaneous neural activity in the visual cortex between the EC and EO states. For example, a lower ALFF in the bilateral visual cortex and higher ALFF in the right PCL were observed in EC compared with EO (Yang et al., 2007). One possible interpretation is that the visual cortex was influenced by visual input during EO (Raichle et al., 2001; Uludag et al., 2004). In contrast, several studies indicated that EC induced greater changes in the spontaneous neural activity in visual regions (Marx et al., 2003, 2004). In addition to these local changes in brain activity, Jao et al. (2013) found that the mean fALFF of the whole brain was significantly higher during EC than during EO.

Considering the widely distributed differences in fALFF between EC and EO and conflicting findings of neural activity changes within the same region, we speculate that the specific pattern of results may depend on the statistical methods. Although several statistical methods (e.g., univariate, bivariate, and multivariate methods) have already been used to detect changes in the fALFF between eye conditions (Yang et al., 2007; Yan et al., 2009; Mcavoy et al., 2012; Jao et al., 2013), to the best of our knowledge, few studies have used multivariate methods to identify and decode discriminatory fALFF patterns with regard to different eye conditions. Of note, considering not all of the changed brain activity regions were directly related to the eyes behavior states, it is necessary to investigate which characteristic changes of the fALFF allow for the accurate discrimination between the EC and EO states.

### fALFF patterns of eye conditions

The advantages of multivariate methods have been demonstrated in many previous studies (for review, see Norman et al., 2006). Using SVM methods, neural activity patterns have been identified in fMRI data that allow for the classification of different cognitive tasks (Poldrack et al., 2009), various diseases (Craddock et al., 2009; Poldrack et al., 2009; Shen et al., 2010), and brain aging (Vergun et al., 2013). Inspired by these previous observations, we used a multivariate method, SVM, based on a nonparametric test approach (Chen et al., 2011), to detect the relationship between the whole brain fALFF and the eye conditions (for details, see “Materials and methods”). The present study found that differences in the fALFF between the EC and EO states were observed in regions associated with the DMN but that the pattern of changes across these two conditions was inconsistent for this region (Table 1). In addition to the visual cortex and DMN regions, we also observed widespread regions, including the sensorimotor cortex, basal ganglia, middle insula, fronto-parietal cortex, and cerebellum, with distinct fALFF values between the EO and EC states (Figure 3). Our results are consistent with those of previous studies, in which a number of distributed regions were affected by eye conditions. For example, EO activated ocular motor and attentional systems associated with “exteroceptive” mental states (Marx et al., 2004), whereas EC activated the visual, somatosensory, and auditory systems and caused significantly higher fALFF in posterior brain regions associated with “interoceptive” mental states (Marx et al., 2004).

Although changes in the fALFF were observed in many regions, not all the changes could be used to discriminate between the eye conditions. In the present study, we found that the fALFF in the sensorimotor cortex was a significant contributor to the whole-brain fALFF map of EC compared with EO. This finding is consistent with a previous study, which showed that brain regions associated with sensory processing were easily influenced by the eye behavior states (Marx et al., 2003).

Moreover, we found that the classifier performance was similar for the fALFF patterns corresponding to *Z* > 1.96 and |*Z*| > 1.96 but that the classifier performance was much lower for the fALFF pattern corresponding to *Z* < −1.96. We found that sensorimotor regions composed approximately 86% of all regions in the fALFF pattern of *Z* > 1.96. The statistical analysis indicated that fALFF was significantly higher in sensorimotor regions in the EC state than in the EO state (Figure 4). There exists a possibility that EO leads to a suppression of sensory modalities to allocate resources to exteroceptive processes (Xu et al., 2014). In the current study, we further suggest that this neurological process specific to sensorimotor regions may play a vital role in the predicting of mental states.

### Eye conditions and mental states

Although exteroceptive and interoceptive states might not be directly manipulated by eye conditions, the current results and previous findings have demonstrated that EO and EC correspond to these two states and that each is associated with distinct brain activity patterns (Marx et al., 2003, 2004). Consistent with studies that involved interoceptive manipulations (Critchley et al., 2004; Simmons et al., 2013), sensorimotor regions including the insula, fronto-parietal regions, and precentral gyrus showed higher spontaneous activity during EC than during EO (Table 1). Activity in sensorimotor regions was previously observed to be associated with mental imagery during a resting state (Mazard et al., 2002, 2005; Assaf et al., 2006). Wang et al. (2008) found that spontaneous resting activity of the sensorimotor cortex was associated with that of the primary visual cortex when subjects remained in the EC state, suggesting that mental imagery occurs in the resting brain in the absence of tasks. Because sensorimotor activity patterns can accurately differentiate between these two states, the results of the present study suggest that there may be a unique mental state associated with the resting state during EC compared with task-dependent conditions and the EO resting state. The neural ensemble of the resting state during EC may provide an approach to explore the neural mechanisms of interoceptive states.

### Limitations

This study contains several limitations that should be addressed in future studies. First, we concentrated on the amplitude of brain spontaneous fluctuations, and explored its association with two resting state conditions (i.e., EC and EO) with the help of the SVM in the present study. Other dimensions of spontaneous activity, such as functional connectivity and network topological organization of spontaneous activity, should also be considered in future studies. Second, the whole-brain fALFF map was constructed based on 160 ROIs derived from a previous meta-analysis. It may be an interesting topic in future research to investigate whether the findings of the present study were dependent on the spatial resolution of the fALFF (e.g., voxel-wise, and AAL-90 or AAL-1024). Third, for the reason that the EC and EO conditions provide EEG measures differing in topography as well as power levels (Barry et al., 2007, 2009), the influence of the frequency band should be further considered in the investigation of the neural substrate underlying EC and EO.

## Conclusion

In summary, the present study investigated the associations between the amplitude of brain spontaneous fluctuations and the EO and EC conditions using a machine learning method. We found a direct relationship between different eye conditions and the fALFF during a resting state, in which most of the regions were located in the sensorimotor cortex. The fALFF changes (increases and decreases) in these sensorimotor regions were significantly modulated by the eye behavior states. Our findings may provide new insights into the neural substrate of distinct resting state activity associated with different eye states.

### Conflict of interest statement

The authors declare that the research was conducted in the absence of any commercial or financial relationships that could be construed as a potential conflict of interest.
